# Family practice research in the African region 2020–2022

**DOI:** 10.4102/phcfm.v16i1.4329

**Published:** 2024-02-13

**Authors:** Robert J. Mash, Klaus von Pressentin

**Affiliations:** 1 Division of Family Medicine and Primary Care, Faculty of Medicine and Health Sciences, Stellenbosch University, Cape Town, South Africa; 2 Division of Family Medicine, Faculty of Medicine, University of Cape Town, Cape Town, South Africa

**Keywords:** family medicine, family practice, primary care, primary health care, research, health services research, clinical research, primary care research, Africa

## Abstract

**Background:**

The African region produces a small proportion of all health research, including primary health care research. The SCOPUS database only lists the African Journal of Primary Health Care & Family Medicine (PHCFM) and the South African Family Practice Journal (SAFP) in the field of family practice.

**Aim:**

To review the nature of all original research (2020–2022) published in PHCFM and SAFP.

**Setting:**

African region.

**Method:**

All 327 articles were included. Data were extracted into REDCap, using a standardised tool and exported to the Statistical Package for Social Sciences.

**Results:**

The median number of authors was 3 (interquartile range [IQR]: 2–4) and institutions and disciplines 1 (IQR: 1–2). Most authors were from South Africa (79.8%) and family medicine (45.3%) or public health (34.2%). Research focused on integrated health services (76.1%) and was mostly clinical (66.1%) or service delivery (37.9%). Clinical research addressed infectious diseases (23.4%), non-communicable diseases (24.6%) and maternal and women’s health (19.4%). Service delivery research addressed the core functions of primary care (35.8%), particularly person-centredness and comprehensiveness. Research targeted adults and older adults (77.0%) as well as health promotion or disease prevention (38.5%) and treatment (30.9%). Almost all research was descriptive (73.7%), mostly surveys.

**Conclusion:**

Future research should include community empowerment and multisectoral action. Within integrated health services, some areas need more attention, for example, children, palliative and rehabilitative care, continuity and coordination. Capacity building and support should enable larger, less-descriptive and more collaborative interdisciplinary studies with authors outside of South Africa.

**Contribution:**

The results highlight the strengths and weaknesses of family practice research in Africa.

## Introduction

Although the quantity and quality of research from sub-Saharan Africa is increasing, the output is still less than 1% of the world’s research.^1^ This is also true of health science research on the continent, with the global contribution increasing to just 1.3% in 2014.^2^ Within the health sciences, the amount of research on family practice and primary healthcare is thought to be disproportionately low.^3^ Recently, there have been calls for 30% of donor funds to help strengthen health systems and service delivery, rather than just focusing on vertical disease programmes.^4^

Primary health care is known to be the one part of the health system that can reduce costs while also improving health status and equity.^5^ Governments in Africa recommitted themselves to implement primary health care in the 2018 Astana Declaration and again at the United Nations General Assembly in 2023.^6,7^ Subsequently, the World Health Organization (WHO) has published both an operational plan and a measurement framework.^8,9^ Family medicine is a medical discipline dedicated to primary health care and district health services in the African context.^10^

Unfortunately, primary health care remains underdeveloped in most African countries.^11^ Service coverage is improving, but is still lower than any other region of the world.^12^ Out-of-pocket spending on health exceeds 20% of all health expenditure in 36 of 47 African countries.^13^ Most countries have yet to meet the minimum threshold for 80% skilled birth attendance, reflecting the low density of nurses and doctors.^14^ Most African countries do not reach the target of 15% of government expenditure on health and 75% of African countries spend less than the requirement of $127.00 per person to deliver an essential package of health services.^15^

Research is needed to support the development of primary health care in Africa and to realise the Astana Declaration. The Declaration specifically addresses the need for such knowledge and capacity building by stating:

We will apply knowledge, including scientific as well as traditional knowledge, to strengthen PHC, improve health outcomes and ensure access for all people to the right care at the right time and at the most appropriate level of care, respecting their rights, needs, dignity and autonomy. We will continue to research and share knowledge and experience, build capacity and improve the delivery of health services and care.^6^

There is therefore a need to analyse the state of family practice research as a lens on the broader state of primary health care research in the region. In this review, the two editors-in-chief of the *African Journal of Primary Health Care & Family Medicine* (PHCFM) and the *South African Family Practice* (SAFP) decided to analyse all original research that was published between 2020 and 2022. The concept was based on a prior analysis of the work published in the SAFP.^16^ It was noted that in the African context, these two journals are the only ones listed in the subject area of family practice (which includes primary health care) by the SCOPUS database.^17^ SCOPUS selects journals for its database based on a number of criteria: a publicly described peer review process, regular publication, content that is relevant and readable for an international audience and a publicly available policy on publication ethics and malpractice.^18^ The aim was to review the state of family practice research in the African region through the publications in the PHCFM and SAFP journals.

## Methods

### Study design

This was a review of original research articles published in the SAFP and PHCFM journals between 2020 and 2022.

### Setting

The PHCFM journal was established in 2009 by the PRIMAFAMED (primary care/family medicine) network to provide an open access platform for researchers in the fields of family medicine and primary health care.^19^ PRIMAFAMED is a network of departments of family medicine and primary health care in sub-Saharan Africa, and the journal was started with funding obtained by this network. The journal is published by AOSIS and depends on article processing charges (APCs) from the authors. It is a regional journal and only publishes research conducted in Africa. It is also the official journal of WONCA Africa. The PHCFM mainly focuses on publishing original research articles.

The SAFP was founded in 1980 as the official journal of the South African Academy of Family Physicians (SAAFP) and primarily publishes material from Southern Africa.^20^ It focuses on research relevant to family medicine, primary health care (PHC) and district health services. It is an open access journal and publishes a range of article types to meet the needs of the family medicine community and members of the SAAFP. It is also published by AOSIS and requires authors to pay APCs.

**FIGURE 1:**
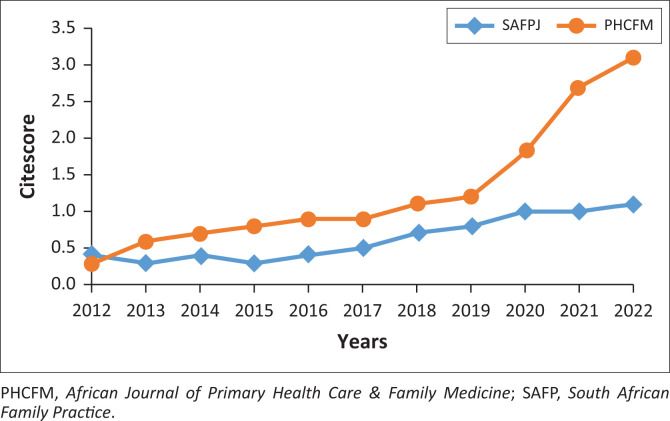
SCOPUS CiteScore for South African Family Practice Journal and African Journal of Primary Health Care & Family Medicine 2012–2022.

Both journals have gradually increased their impact over the last 10 years as shown by the increasing SCOPUS CiteScores in Figure 1.^17^

### Selection of journals and articles

These two journals were selected from the SCOPUS database as the only two African journals listed under the subject area of ‘family practice’.^17^ SCOPUS lists 47 journals in this subject area and PHCFM is ranked 15/47 and SAFP as 27/47. In the African region, therefore, they are the two leading journals for the discipline of family medicine. All original research articles (including structured reviews and scientific letters) were selected for inclusion in the analysis.

### Extraction of data

**FIGURE 2:**
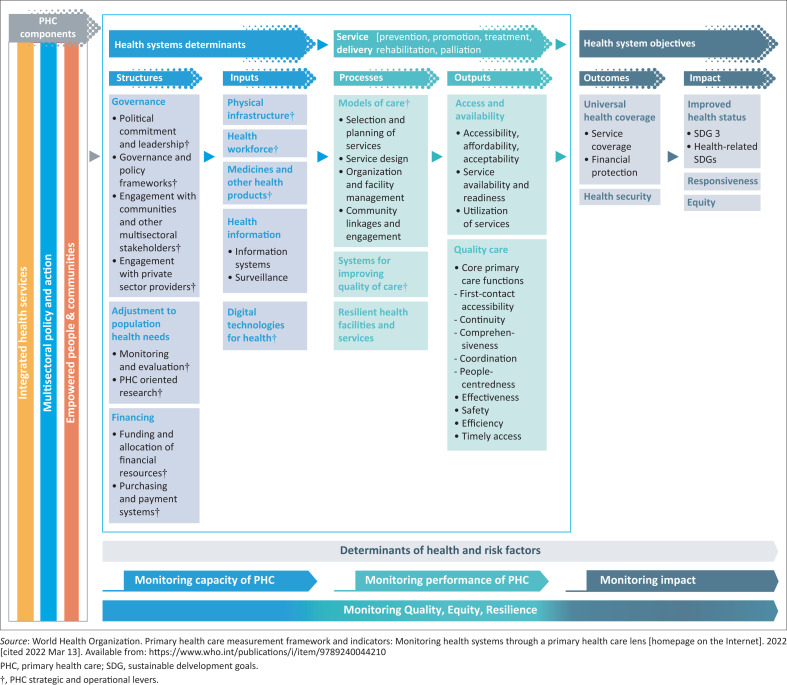
Primary health care monitoring conceptual framework.

A number of frameworks were used to guide data extraction. These included the three components of PHC as per the World Health Organization’s (WHO) operational guideline: integrated health services, community empowerment and multisectoral policy and action.^8^ The typology of primary care research was also used to categorise the studies: basic research, clinical, health services, health systems and educational.^21^ In addition, the WHO measurement framework for PHC was used to deconstruct the focus of health systems and health services research as per the subdomains shown in Figure 2.^9^

R.J.M. extracted data from the PHCFM and K.v.P. from the SAFP. A structured data collection tool was jointly developed in REDCap for this purpose and included the following fields:

- Year of publication.
- Country affiliation of the first author and all authors.
- Total number of authors per article.
- Total number of institutions and disciplines listed per article.
- Disciplinary backgrounds of the authors.
- Focus of article according to the WHO’s PHC components.
- Focus of article according to the typology of primary care research.
- Type of study design.
- Focus of clinical articles in terms of disease categories.
- Focus of articles in terms of the life course.
- Focus of articles in terms of the continuum of care.
- Focus of health systems and health services research on subdomains of the WHO measurement framework.
- Focus of educational research.

### Data analysis

The data were downloaded from REDCap and imported into SPSS^®^ for analysis. Data were analysed descriptively by the two authors. Categorical data are presented as frequencies and percentages. Numerical data were not normally distributed and are presented as medians and interquartile ranges (IQRs). Data from both journals are combined, as the intention was not to compare the two journals but represent the research output as a whole.

### Ethical considerations

This article followed all ethical standards for research without direct contact with human or animal subjects.

## Results

Overall, 327 research articles were published, with 196 (59.9%) from the PHCFM and 131 (40.1%) from SAFP. Publications were evenly divided between the 3 years, with 2020 (*n* = 107, 32.7%), 2021 (*n* = 108, 33.0%) and 2022 (*n* = 112, 34.3%). The median number of authors per article was 3 (IQR: 2–4), although the median number of institutions and disciplines per article was both 1 (IQR: 1–2).

The majority of the first authors came from South Africa (*n* = 240, 73.4%) as shown in Table 1. The PHCFM journal had a higher proportion of first authors from outside of South Africa (36.7% vs. 11.5%). After South Africa, only Nigeria had a substantial number of first authors (*n* = 25, 7.6%). This pattern is repeated, when all authors are considered, as shown in Table 1. Authors from outside of Africa came from the United States (*n* = 16, 4.9%), United Kingdom (*n* = 8, 2.4%), Australia (*n* = 5, 1.5%), Belgium (*n* = 4, 1.2%), Malaysia (*n* = 2, 0.6%) and Germany (*n* = 1, 0.3%). All of the authors from outside of Africa were in support of Africans as first authors. Some African countries, such as Mozambique and Zambia, had no authors represented.

**TABLE 1:** Country origins of first and all authors (*n* = 327).

Country	First authors		All authors	
	*n*	%	*n*	%
South Africa	240	73.4	261	79.8
Nigeria	25	7.6	30	9.2
Ghana	8	2.4	9	2.8
DR Congo	6	1.8	6	1.8
Malawi	8	2.4	8	2.4
Botswana	4	1.2	4	1.2
Egypt	4	1.2	4	1.2
Namibia	3	0.9	3	0.9
Kenya	4	1.2	10	3.1
Zimbabwe	3	0.9	3	0.9
Eswatini	2	0.6	2	0.6
Lesotho	2	0.6	3	0.9
Uganda	1	0.3	1	0.3
Tanzania	1	0.3	3	0.9
Ethiopia	1	0.3	1	0.3
Gambia	1	0.3	1	0.3
Morocco	1	0.3	1	0.3
Rwanda	0	0.0	1	0.3
Cameroon	0	0.0	1	0.3
Sierra Leone	0	0.0	1	0.3

Table 2 shows the disciplines of the authors. The majority of articles had authors from family medicine (45.3%) and public health (34.2%). The SAFP had a higher proportion of articles with authors from family medicine (55.7% vs. 38.3%). There was a broad range of disciplines including nursing and midwifery (16.8%), psychiatry and psychology (7.9%), educationalists (6.7%), social sciences (6.4%) and biostatistics (5.2%).

**TABLE 2:** Authors’ disciplinary backgrounds (*n* = 327).

Disciplines	*n*	%
Family medicine	148	45.3
Public health	112	34.2
Nursing and midwifery	55	16.8
Internal medicine	27	8.2
Psychiatry and psychology	26	7.9
Health science education	22	6.7
Social science	21	6.4
Biostatistics	17	5.2
Obstetrics and gynaecology	16	4.9
Emergency medicine	13	4.0
Pharmacy and pharmacology	12	3.7
Child health and paediatrics	10	3.1
Management and administration	9	2.8
Physiotherapy	8	2.4
Pathology	7	2.1
Surgery	6	1.8
Environmental science	4	1.2
Optometry and ophthalmology	3	0.9
Speech therapy and audiology	3	0.9
Anaesthesia	2	0.6
Occupational therapy	1	0.3
Oral health and dentistry	1	0.3

The overall focus of the research in terms of the key components of PHC was on integrated health services (76.1%) (Table 3). Although 12.2% of the research focused on empowered people and communities, this was mostly community-based surveys of community health needs and not specifically on community empowerment. There was no focus on multisectoral policy and action (0.3%). The majority of the research was clinical (66.1%), with a strong secondary focus on service delivery (37.9%). There was much less of a focus on basic, health systems and educational research. Almost all of the research, whether quantitative or qualitative in approach, was descriptive in nature (73.7%). There was very little observational or experimental research. Much of the research focused on adults or older adults (77.0%), with a secondary focus on adolescents and pregnant women. There was little focus on children, infants or neonates. In terms of the continuum of care, there was a strong focus on health promotion and disease prevention (38.5%) and treatment (30.9%). However, there was little focus on palliative care or rehabilitation.

**TABLE 3:** Focus of research (*n* = 327).

Research focus areas	*n*	%
**Components of primary health care**		
Multisectoral policy and action	1	0.3
Integrated health services	249	76.1
Empowered people and communities	40	12.2
Other	22	6.7
**Typology of research**		
Basic	3	0.9
Clinical	216	66.1
Service delivery	124	37.9
Health systems	41	12.5
Educational	39	11.9
Other	1	0.3
**Type of study design**		
Descriptive or cross-sectional survey	181	55.4
Descriptive exploratory qualitative	60	18.3
Mixed methods	26	8.0
Scoping or systematic review	14	4.3
Experimental/clinical trial	8	2.4
Phenomenology	9	2.8
Observational analytical	7	2.1
Quality improvement	1	0.3
Participatory action research	1	0.3
Other	20	6.1
**Focus on the life course**		
Adult	196	59.9
Older adult	56	17.1
Pregnant woman	27	8.3
Adolescent	32	9.8
Child	21	6.4
Infant	10	3.1
Neonate	4	1.2
Not applicable	14	4.3
Not specific/all age groups	54	16.5
**Focus on the continuum of care**		
Health promotion	39	11.9
Disease prevention	87	26.6
Curative/treatment	101	30.9
Rehabilitation	6	1.8
Palliation	7	2.1
Not applicable	87	26.6

Table 4 breaks down the focus of the research according to the typology. For clinical research, there was a substantial focus (67.4%) on infectious diseases, non-communicable diseases as well as maternal and women’s health. There was much less published on mental health, trauma and violence.

**TABLE 4:** Specific focus areas within the research typology.

Type of research	*n*	%
**Clinical focus (*n* = 248)**		
Non-communicable diseases	61	24.6
Infectious diseases (e.g. HIV, TB, malaria, COVID)	58	23.4
Maternal and women’s health	48	19.4
Mental health or substance abuse	29	11.7
Intentional trauma (e.g. abuse, IPV)	12	4.8
Unintentional trauma (e.g. RTA)	7	2.8
Other	33	13.3
NA	-	-
**Service delivery focus (*n* = 176)**		
Core primary care functions	63	35.8
Service design	24	13.6
Access, availability and utilisation	21	11.9
Safety of patients or staff	12	6.8
Systems for quality improvement	17	9.7
Community engagement and linkages	21	11.9
Organisation and facility management	6	3.4
Resilience of health facilities and services	8	4.5
Effectiveness	2	1.1
Efficiency	2	1.1
**Function (*n* = 69)**		
First contact access	5	7.2
Continuity	1	1.4
Coordination	5	7.2
Comprehensiveness	22	31.9
Person-centredness	36	52.2
**Type of health systems research (*n* = 44)**		
Governance and policy	5	11.4
Health financing	4	9.1
Infrastructure	3	6.8
Medication, equipment and supplies	5	11.4
Workforce	16	36.4
Health information system	3	6.8
Digital technology	8	18.2
**Type of educational research (*n* = 41)**		
Undergraduate	18	43.9
Postgraduate	9	22.0
In-service/CPD	12	29.3
Other	2	4.9

HIV, human immunodeficiency virus; TB, tuberculosis; COVID, corona virus infectious disease; IPV, intimate partner violence; RTA, road traffic accident; CPD, continuous professional development.

Health services research mainly focused on the core primary care functions (35.8%), with a secondary focus (37.4%) on service design or the model of care, as well as access, availability, utilisation and community engagement or linkages. There was very little research on facility organisation and management, effectiveness, efficiency and the resilience of facilities and services. In terms of the five core functions of primary care, the focus was mostly on person-centredness and comprehensiveness of care (84.2%). There was very little research on first contact access, continuity and coordination of care.

Health systems research mainly focused on the workforce (36.4%) and use of digital technology (18.2%). There was little attention given to infrastructure, health information systems, equipment and supplies, as well as overall governance, policy and financing.

Educational research was mostly on undergraduate programmes, followed by continuing professional development and lastly postgraduate programmes.

## Discussion

### Summary of key findings

Research focused on integrated health services and was mostly clinical or focused on aspects of service delivery. Clinical research mainly addressed infectious diseases, non-communicable diseases and maternal and women’s health. Service delivery or health services research mainly addressed the core functions of primary care, particularly person-centredness and comprehensiveness. Research mostly targeted adults and older adults as well as health promotion, disease prevention and treatment. Almost all of the research was descriptive in nature and the majority of publications involved surveys. The majority of authors were from South Africa and from either family medicine or public health. Most articles involved only one institution and one discipline. Nevertheless, the work is growing in its impact, as judged by steadily increasing SCOPUS CiteScores.^17^

### Discussion of key findings

Implementation of PHC requires evidence on how to engage communities as well as collaborate with different sectors, such as social services and education.^8^ This enables a broader focus on the social and environmental determinants of health, as well as population health management. In Africa, a community-orientated model of primary care is promoted,^22^ and family physicians can help lead implementation.^23^ This community-orientated model includes an emphasis on community engagement, multisectoral collaboration and proactively reaching out to the whole population at risk. Primary care is, therefore, integrated with essential public health functions as envisaged by the WHO.^8^ The findings suggest that researchers need to have a broader approach and move from primary care research to PHC research.

Although mental health problems are a major contributor to morbidity in Africa,^24^ they have been largely neglected in this research agenda. This may be because donor funding is driven by mortality data and the burden of disease, but may also reflect the relative neglect of primary mental healthcare as part of comprehensive primary care. The WHO suggests that primary care should routinely address depression, anxiety disorders, alcohol and substance abuse, sleep problems, chronic tiredness and unexplained somatic complaints and not just see mental healthcare as focused on severe disease and psychotic disorders.^25^ Likewise, intentional and unintentional traumas are not researched and this may reflect the difficulty of engaging complex social and environmental determinants of trauma from intimate partner violence, community violence and conflict, as well as road traffic accidents. Addressing mental health, violence and trauma necessitates breaking out of the medical model and looking at broader multisectoral and community issues.

Among the core functions of primary care, the focus on person-centredness included studies that reflected the perspectives of users and their satisfaction with health services as well as a more specific focus within the consultation on patient’s perspectives. Continuity of care has a powerful impact on improved hospitalisation rates and clinical outcomes,^26,27^ yet this core function was under-researched. Informational continuity links strongly to health information systems and patient medical records, which were also rarely addressed. Relational continuity of care is known to be a weakness of African PHC and researchers may feel that this is not a feasible goal with the scarce human resources and lower trained cadres. Likewise, coordination is a key aspect of quality care and related to a more functional information system,^27^ yet appears to be an unusual focus for researchers in the family practice subject area.

Leadership and organisational management at the level of the facility and district is key to improving the quality of care and recognised as a weakness of many health systems.^28^ Leadership needs to become more collaborative and appreciative, with more decentralised authority, and the ability to experiment, plan and innovate. There was, however, little research on this topic and this may reflect the clinical focus of many researchers.

It is perhaps not surprising that health systems research was less common, as family practice is more concerned with service delivery and clinical care. The adequacy of the workforce in terms of numbers, composition, competency and team-based care was a strong theme and in particular the contribution of family physicians to district health services. The emergence and development of family medicine across the region has been a focus of both journals.

As Africa faces new climate-related challenges and the likelihood of further pandemics,^29^ the preparedness of health facilities and services becomes more important.^30^ The WHO has recognised the importance of this in their measurement subdomain on resilience – the ability to continue providing services, respond to challenges and bounce back.^9^This is an area of research that should receive more attention in the future.

Research capacity in Africa, particularly in the field of family practice, is thought to be low and often driven by students completing Master’s degree.^31^ This may partly explain the focus on descriptive study designs and surveys. Researchers may also lack the capacity to compete for international funding that would enable them to engage with larger scale observational and experimental study designs. This would also allow more studies that address effectiveness and efficiency. It is also possible that these types of studies were published in more international journals, with a higher impact, if they do exist. The PRIMAFAMED network has noted the need to develop established researchers through the pursuit of doctoral degrees and collaborations.^31^

Other areas that appear to be neglected include research on children. This might partly be because of the increased difficulty of researching this age group, particularly in obtaining ethics approval. The focus on health promotion and disease prevention was surprisingly strong. Palliative care is an emerging priority in several countries and often supported by the discipline of family medicine.^32^ However, research on palliative care was still low. Rehabilitation in family practice and primary healthcare is another weakness within the health services and the lack of research may reflect the lack of focus on physical medicine and rehabilitation within family practice.

There is clearly a need to foster research outputs from many countries in the region and to balance the dominance of family practice research from South Africa. African research as a whole follows a similar pattern.^33^ Although there were many authors coming from outside Africa, they mostly supported publications by African researchers as first authors. The PHCFM journal has also been found to have the highest proportion of African women as first authors within the BMJ health research database.^34^ French- and Portuguese-speaking countries may prefer to publish elsewhere or struggle with writing in English. For many low- and low-middle-income countries, the APCs may prohibit submissions, even though these are lower than international journals and waivers are also possible. Much of the research driven by Master’s degrees may not be publishable, especially if supervisors are also lacking capacity in postgraduate supervision. South Africa is fortunate in that the government provides a subsidy for research publications to researchers. More interdisciplinary and collaborative research projects would also help with exploring neglected areas and building research capacity.

### Strengths and limitations

The findings represent the research published in these two journals and may not be representative of all research published by family practice researchers. Although these are the two leading African journals in the subject area, many authors may choose to publish in more international (e.g. BMC Primary Care), general medical (e.g. PloS One) or more discipline-specific journals (e.g. African Journal of Disability). There are also many national or sub-regional journals that are not listed in databases such as SCOPUS (e.g. Lesotho Medical Journal) or have not generated a CiteScore (e.g. East African Medical Journal). The inclusion of the SAFP will have inflated the proportion of authors from South Africa. Nevertheless, the findings give some indication of the state of family practice and PHC research.

It was difficult to determine the disciplinary background of some authors, as the publishers usually record only the name of the department and institution. In some instances, the researchers searched the internet for more information on the specific author. We had hoped to identify whether research was derived from students in degree programmes, but this information was not consistently recorded in the acknowledgements and was not reliable.

### Implications

The findings suggest the following:

- Researchers in the field of family practice and PHC should look at research questions related to community engagement and empowerment as well as multisectoral action, to fully address implementation of PHC.
- Researchers should consider areas that appear to be under-researched, such as children, palliative care, rehabilitation, mental health, trauma and violence, as well as continuity and coordination of care. Additional areas to consider are leadership, organisational management and the future resilience of facilities and services.
- Strategies are needed to build PHC research capacity in countries outside of South Africa. The PRIMAFAMED network and initiatives such as the Afriwon Research Collaboration are addressing this issue, but more is needed at national and regional levels. Strategies need to address the whole pipeline from novice to established researchers and develop postgraduate supervisory capability. More doctoral-level research training would assist with this and dedicated funding opportunities for PHC research. Assistance is also needed to afford the APCs in open access journals, which are often prohibitive for researchers in Africa.
- Strategies are also needed to encourage collaborative research between institutions and disciplines that enable larger scale projects and capacity building and address more complex issues such as mental health, violence and trauma.

## Conclusion

African research, published in the field of family practice in these two leading journals, has both strengths and weaknesses. On the one hand, there is growing impact and a strong focus on both clinical and service delivery-related research. On the other hand, there is little attention given to community engagement and empowerment as well as multisectoral action. A number of important areas appear to be neglected, such as children, palliative care, rehabilitation, mental health, trauma and violence, as well as continuity and coordination of care. Researchers from countries outside of South Africa need support to build capacity and to overcome the barriers to publication in open access journals. Strategies are needed to encourage more interdisciplinary and collaborative research.
